# Tranylcypromine Causes Neurotoxicity and Represses BHC110/LSD1 in Human-Induced Pluripotent Stem Cell-Derived Cerebral Organoids Model

**DOI:** 10.3389/fneur.2017.00626

**Published:** 2017-12-07

**Authors:** Jing Huang, Fangkun Liu, Hui Tang, Haishan Wu, Lehua Li, Renrong Wu, Jingping Zhao, Ying Wu, Zhixiong Liu, Jindong Chen

**Affiliations:** ^1^Department of Psychiatry, The Second Xiangya Hospital, Central South University (CSU), Changsha, China; ^2^Mental Health Institute of the Second Xiangya Hospital, Chinese National Technology Institute on Mental Disorders, Central South University (CSU), Chinese National Clinical Research Center on Mental Disorders, Hunan Key Laboratory of Psychiatry and Mental Health, Changsha, China; ^3^Department of Neurosurgery, Xiangya Hospital, Central South University (CSU), Changsha, China; ^4^Intensive Care Unit, The Second Xiangya Hospital, Central South University (CSU), Changsha, China

**Keywords:** cerebral organoids, tranylcypromine, neurotoxicity, *in vitro* models, neuropsychiatric disease

## Abstract

Recent breakthroughs in human pluripotent stem cell-derived cerebral organoids provide a valuable platform for investigating the human brain after different drugs treatments and for understanding the complex genetic background to human pathology. Here, we identified tranylcypromine, which is used to treat refractory depression, caused human-induced pluripotent stem cell-derived brain organoids neurotoxicity, leading to decreased proliferation activity and apoptosis induction. Moreover, tranylcypromine treatment affects neurons and astrocytes, which impairs cell density and arrangement. Finally, staining of histone demethylation-related genes revealed that tranylcypromine suppresses the transcriptional activity of BHC110/LSD1-targeted genes and increases the expression of histone di-methylated K4. These results show that human brain organoids can be applied as an *in vitro* model for CNS drug screening to evaluate structural, cellular, and molecular changes in the normal brains or brains of patients with neuropsychiatric disorders after drug treatments.

## Introduction

Depression is a common and severe neuropsychiatric disorder of great societal and medical importance, which contributes greatly to world health problem (1). Drug treatments include monoamine oxidase inhibitors (MAOIs), tricyclic antidepressants, selective serotonin reuptake inhibitors, as well as various drug combinations (2–5). The safety, efficacy, and side effects of different drugs have been extensively studied using current research models (6). However, animal models lack human genetic features and some brain region identities, such as outer subventricular zone, making it hard to figure out how these drugs affect central nervous system (7, 8).

For better disease modeling, toxicology, and drug discovery studies, *in vitro* 3D culture models of the whole organ have been developed for different systems in recent years, such as small intestine (9–11), pancreas (12), kidney (13, 14), pituitary (15), retina (13), and brain (16–18). Recent breakthroughs in human pluripotent stem cells (hPSCs)-derived cerebral organoids offer a promising approach for investigating the mystery of human brain (19). Compared with conventional mice model, this model can recapitulate the human brain development *in vitro* (20). The cerebral organoids, which can mimic the endogenous development of human brain, have been applied to different studies as *in vitro* models for a wide range of brain diseases (16, 21, 22). Moreover, 3D human brain organoids show great potential to investigating psychiatric disease origin and pathology, as well as drug screening and genetic modifications (19).

Tranylcypromine (2-PCPA, Parnate), one of nonselective and irreversible MAO-A/MAO-B inhibitors (MAOIs), is used to treat refractory depression after many other drugs have failed to treat the symptoms (3). The common side effects of tranylcypromine are sleep disturbances, orthostatic or postural hypotension, hallucinations, fatigue, blurred vision, headache, and gastrointestinal discomfort and disorders (3, 23, 24). Although with little evidence at the molecular and cellular levels, high-dose tranylcypromine may cause brain damage and inhibit brain development (25–27).

Since models are limited to study the mechanism of the neurotoxicity of tranylcypromine, here we utilized an *in vitro* brain organoid culture system which mimics neurogenesis experimentally to investigate the mechanisms by studying the effect of brain development of tranylcypromine and monitoring the brain damage of different concentrations. BHC110/LSD1 is an enzyme shares close homology with monoamine oxidase (MAO). Tranylcypromine could inhibit demethylase histone by BHC110/LSD1 suppression, thus may decrease the transcriptional activity of BHC110/LSD1-targeted genes (28, 29). Here, we aim to figure out if tranylcypromine can cause brain damage by repressing neuronal-specific genes as well as BHC110/LSD1 target genes. Neuron-specific genes will be detected to see which can be modulated by this anti-depression drug. Finally, BHC110/LSD1 target genes will be examined in different concentrations to further discover the mechanism.

## Materials and Methods

### Cerebral Organoid Culture Procedure

Human-Induced Pluripotent Stem Cells were from System Biosciences (SC101A-1). IPSCs were maintained on irradiated MEFs (MTI-GlobalStem) according to standard protocols (30). When starting the organoid culture, induced pluripotent stem cell (iPSC) colonies were isolated by dispase treatment and trypsinization to obtain the suspension of single cells. 5,000 cells were then plated in each well of 96-well U-bottom plate (Corning Costar) in hESC medium, every 500 ml combining 400 ml of DMEM-F12 (Invitrogen), 100 ml of KOSR (Invitrogen), 5 ml of MEM non-essential amino acid (Sigma), 15 ml of ESC-quality FBS (Gibco), 5 ml of GlutaMAX (Invitrogen), 3.5 µl of 2-mercaptoethanol (Merck), 1× penicillin/streptomycin and 4 ng/ml bFGF (Peprotech).

Medium was changed every other day for 5 days then replaced by neural induction medium, every 500 ml containing 400 ml of DMEM-F12 (Invitrogen), 1× N_2_ Supplement (Invitrogen), 100 ml of KOSR (Invitrogen), 15 ml of ESC-quality FBS (Gibco), 1× GlutaMAX, 1× MEM-NEAA (Sigma), 3.5 µl of 2-mercaptoethanol (Merck), 1× penicillin/streptomycin. On day 8, organoids were maintained in droplets of Matrigel (BD Biosciences) in neural induction medium. On day 11 of the protocol, embedded organoids were plated on Orbital shaker and cultured in cerebral differentiation media, every 500 ml containing 250 ml of DMEM/F12 (Invitrogen) and 250 ml Neurobasal medium (Invitrogen), 1× N_2_ supplement (Invitrogen), 1× B27 supplement (Invitrogen), 3.5 µl/l 2-mercaptoethanol (Merck), 1:4,000 insulin (Invitrogen), 1× glutamax (Invitrogen), 1× MEM-NEAA (Invitrogen), and 1× penicillin–streptomycin (Invitrogen). Organoids were fed every other day by fresh medium. All images were acquired by an inverted microscope.

### Immunostaining and Analysis

Samples were collected and fixed in 10% formalin for 1 h at room temperature followed by embedding in paraffin. Then hemotoxylin/eosin (H&E) staining and immunofluorescence were performed. After blocking, slides were treated with primary antibodies in 5% bovine serum albumin with concentration as follows: anti-N-cadherin (rabbit, Cell Signaling 13116, 1:300), anti-vimentin (rabbit, Cell Signaling 5741, 1:300), anti-Ki-67 (mouse, cell signaling, 1:200), anti-cleaved Caspase 3 (rabbit, Invitrogen, 1:200), anti-Sox2 (mouse, Invitrogen, 1:200), anti-Oct4 (mouse, Invitrogen, 1:200), anti-FoxG1 (rabbit, Abcam, 1:200), anti-TUJ1 (rabbit, Abcam, 1:200), anti-Auts2 (rabbit IgG, Proteintech, 1:50), anti-Islet1 (rabbit IgG, Proteintech, 1:50), anti-Nell2 (rabbit IgG, Proteintech, 1:50), anti-GFAP (rabbit IgG, Abcam, 1:200), anti-H3-di-K4 (rabbit, Abcam, 1:200), anti-LSD1 (rabbit, Life technology, 1:300), anti-CD133 (rabbit, Cell Signaling, 1:300), anti-EGFR (rabbit, cell signaling, 1:300), anti-Tubulin (rabbit, Invitrogen, 1:200), anti-actin (rabbit, Invitrogen, 1:200), anti-nestin (mouse, Invitrogen, 1:200). Secondary antibodies include Alexa Fluor 488 rabbit, Alexa 488 Fluor mouse, Alexa 594 Fluor rabbit, and Alexa 594 Fluor mouse IgG (Invitrogen, 1:500). Tranylcypromine was from Selleck Chemicals. The images of immunostaining were acquired by an Olympus fluorescent microscope.

### Reagents and Cell Viability Assay

The Cell Viability of brain organoids was analyzed by MTT (Sigma) Cell Proliferation Assay. The organoids (1/well) were incubated in a 96-well plate with the indicated concentration of tranylcypromine for 48 h at 37°, 5% CO_2_. Each group used three replicate wells. Then, after treated with 5 mg/ml MTT, the plate was then incubated for 3 h at 37°, and the medium was dumped off and Matrigel was solubilized in 2% SDS. 100 µl of DMSO was added and incubated for 30 min at 37°C, to mix formazan crystals into the solvent. A plate reading spectrophotometer was employed to monitor absorbance at 570 nm. The experiments were performed in triplicates.

### Statistical Analysis

All data are presented as the mean ± SEM. The results with two groups were statistically analyzed by Student’s *t*-test. ANOVA analyses were applied to compare of data with greater than two groups. A value of *p* < 0.05 was considered statistically significant.

## Results

### Generation of Human iPSC-Derived Cerebral Organoids

Human-iPSCs can be differentiated into any human cell identities to be used for disease modeling or drug screening. In 2007, Takahashi et al. described the induction of iPSCs by four defined transcription factors: Oct3/4 (Pou5f1), Sox2, Klf4, and c-myc (31). Recent progress in the generation of 3D cultured organoids *in vitro* from human iPSCs makes it possible to study the brain in a dish (17, 32).

**Figure 1:**
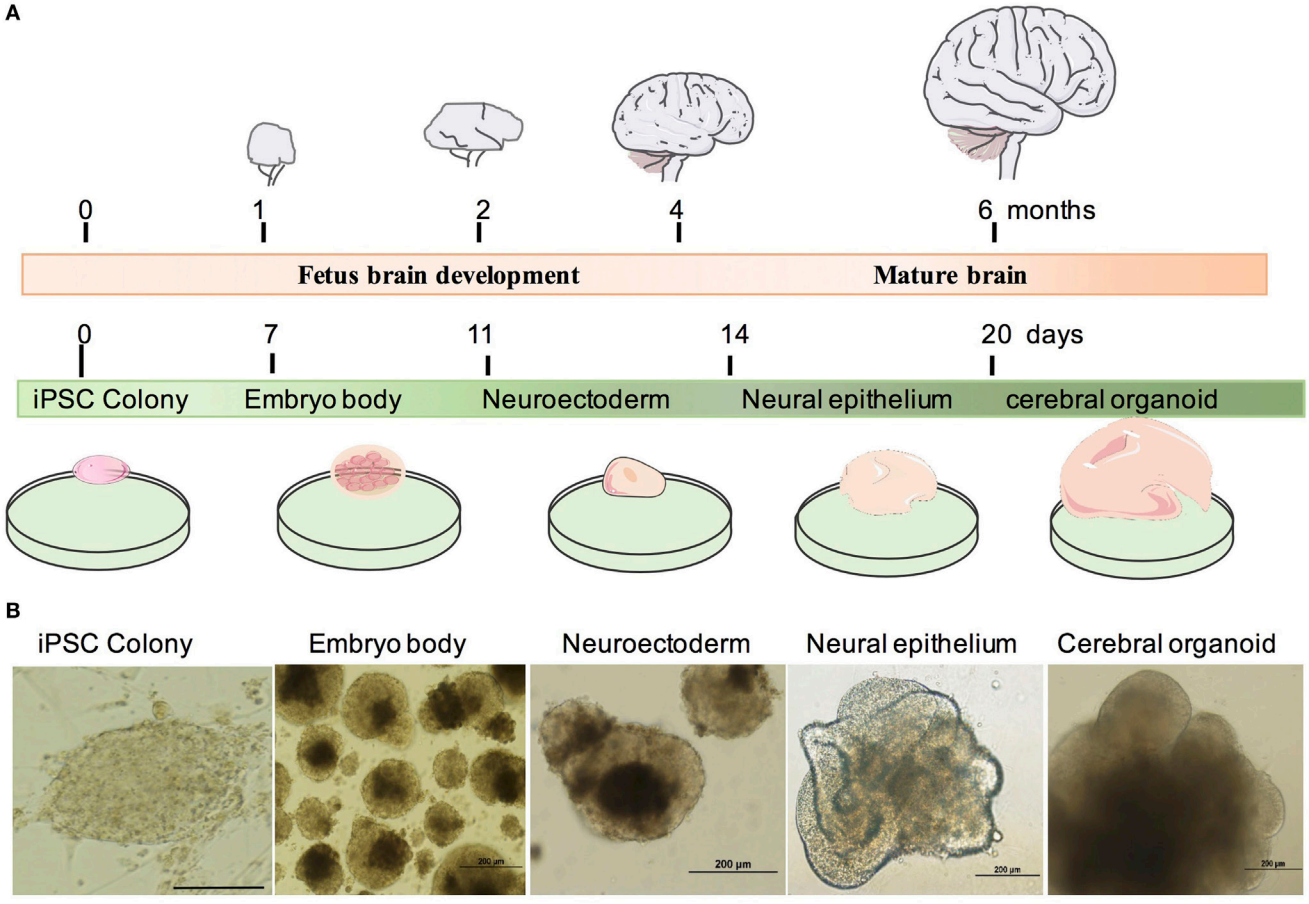
Generation of human human-induced pluripotent stem cell (iPSC)-derived cerebral organoid system. **(A)** Timeline of *in vivo* brain and cerebral organoid development. **(B)** Example of cerebral organoid from human iPSCs. Human iPSCs form embryo bodies, developed into neuroectoderm and finally differentiated into neural epithelium and cerebral organoids. Scale bars: 200 µm.

To study the effect and neurotoxicity of tranylcypromine, we developed an *in vitro* cerebral organoid system from human-iPSCs using the previous protocol (17). The cerebral organoid can be developed by changing the components and culture environments timely and accurately, and recapitulate the fetus brain development *in vivo* (Figure 1A). We, first, made embryoid bodies (EBs) from iPSCs colonies in a six-well plate coated with irradiated MEFs. After 7 days, EBs were then plated in a 24-well plate and supplied with neural induction media to generate neuroectoderm from EBs. The EB has a dark center with dense non-ectodermal tissue and a bright smooth surface tissue which can be differentiated to ectoderm. At day 11, new generated neuroectodermal tissues were transferred to Matrigel droplets to provide a 3D culture for more complex tissue growth. Differentiation media was used in this step. The organoid showed neuroepithelial bud expansion as day 14; then, it was transferred to the orbital shaker for further differentiation. After an average of 20 days, mature cerebral organoid with large neural tissue formed (Figure 1B).

### Cerebral Organoid Tissues Displayed Brain Regions and Neuronal Cell Identities

**Figure 2:**
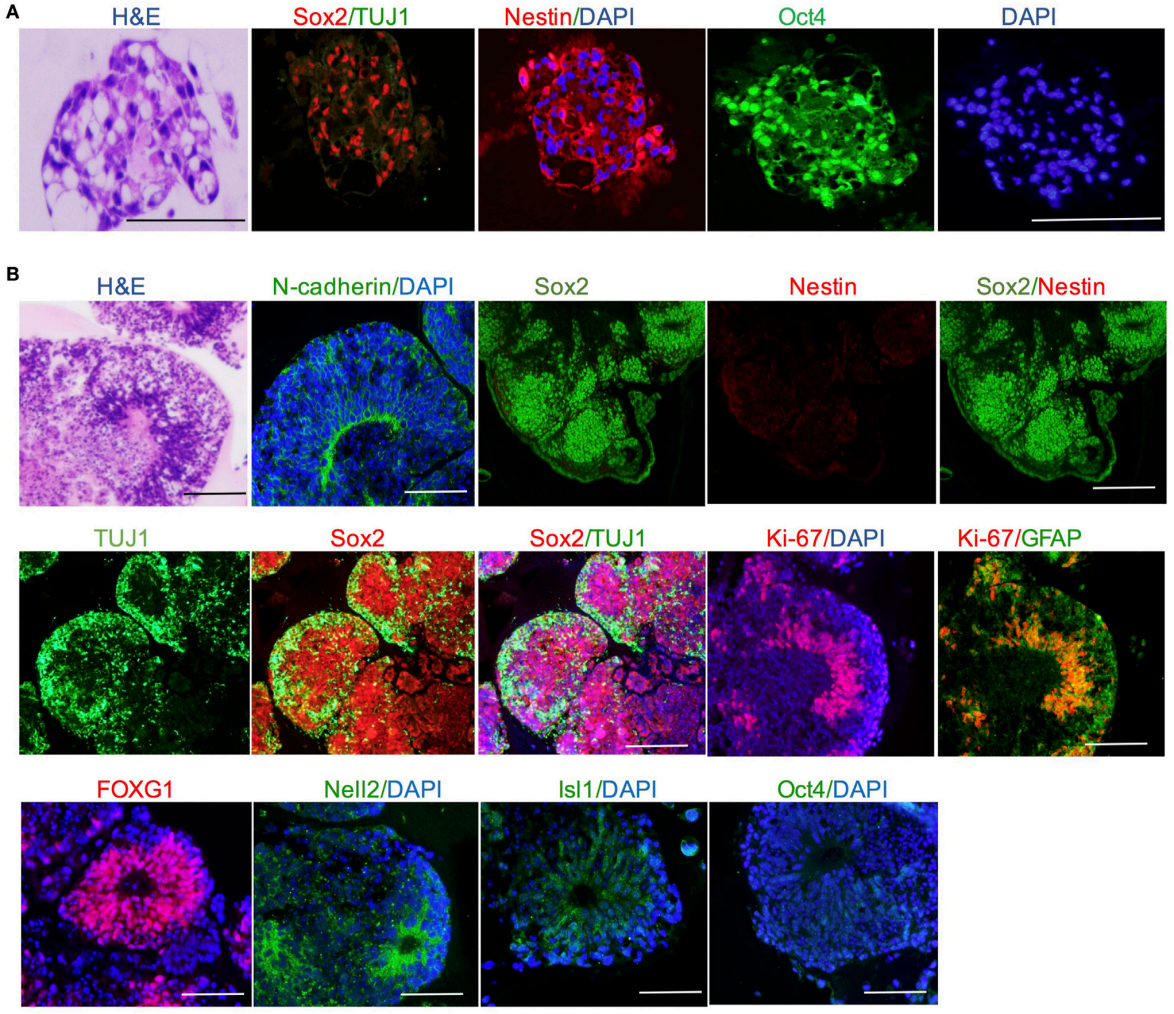
Cerebral organoid tissues displayed brain regions and neuronal cell identities. **(A)** Neuroepithelium at day 11 presents neural precursor differentiation. **(B)** Cerebral organoids at day 20 presented neural N-cadherin in the apical membrane. Forebrain, hindbrain, and hippocampus regions of an organoid stained positive by FOXG1, Nell2, and Isl1 markers, respectively. Neuron-specific cells neurons and glial cells were marked by TUJ1 and GFAP, respectively. Pluripotency markers Oct4 disappeared in mature cerebral organoids. Scale bars: 200 µm.

Histological and morphological analysis was employed to identify brain regions and neuronal cells. At day 11, cerebral organoids show organized neuroepithelium-like structures expressing neural precursor marker Nestin and progenitor marker Sox2, which indicated neural tubes development and radial glial cell differentiation, but stained negative for neuron marker TUJ1 (Figure 2A). At day 20, cerebral organoids exhibited complex morphology and neuron-specific cells differentiation. Histological analysis revealed the complex morphology of a cerebral organoid and suggested it formed heterogeneous brain regions. Forebrain, hindbrain, and hippocampus regions of an organoid stained positive by FOXG1, Nell2, and Isl1 markers, respectively (Figure 2B). Mature cerebral organoids presented neural N-cadherin in the apical membrane surrounding fluid-filled cavities reminiscent of ventricles (Figure 2B). In the cortical tissue within an organoid, Sox2 stained for progenitors and TUJ1 stained for the neurons. All glial cells were marked by GFAP (green) (Figure 2B). For deeper examination, we tested the efficiency of neural induction in organoids and detected the loss of pluripotency marker Oct4 during the course of organoid differentiation due to neural differentiation (Figure 2B).

### Tranylcypromine Affected Cellular Proliferation and Apoptosis in Human Brain Organoids

**Figure 3:**
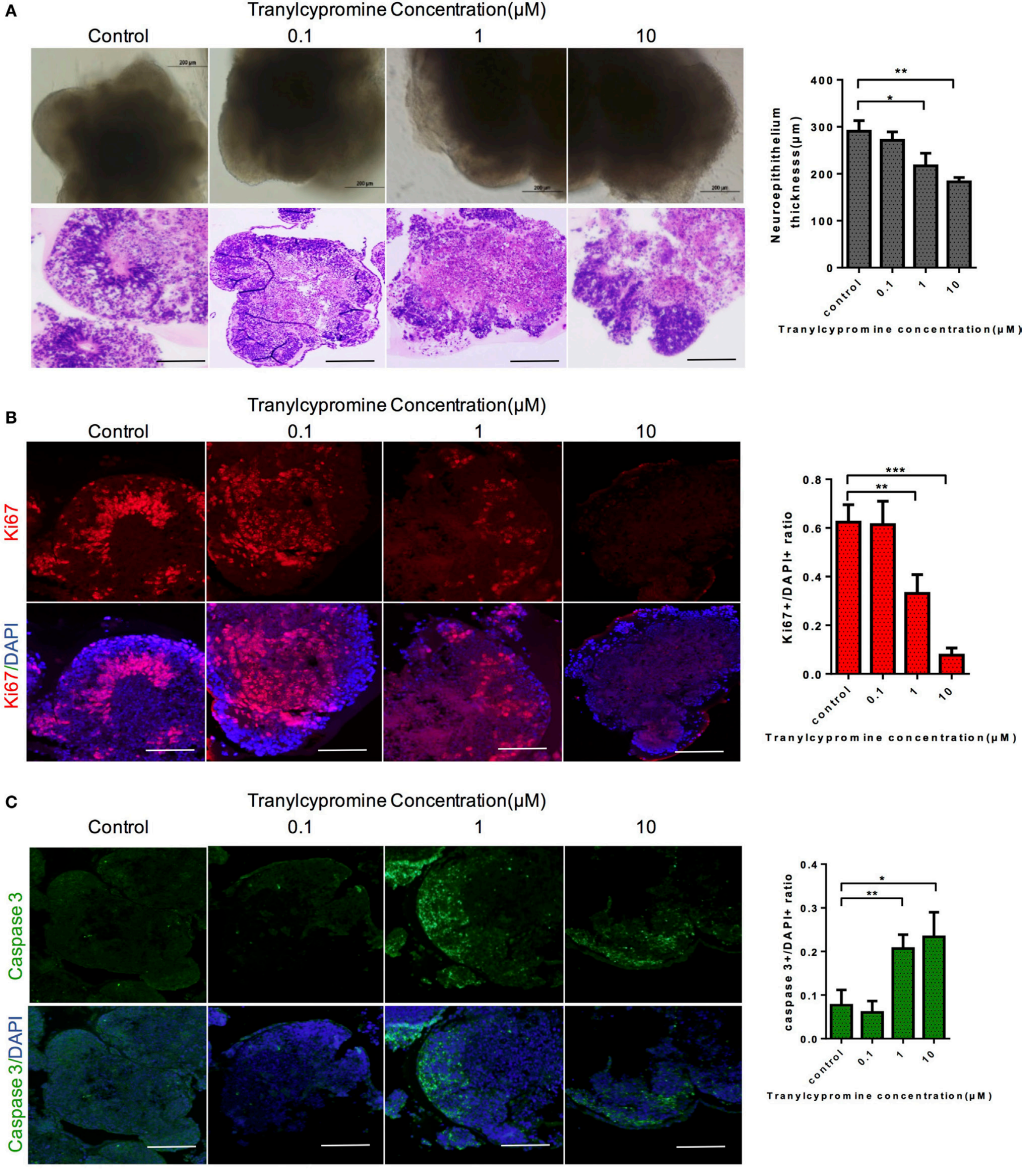
Tranylcypromine affected on cellular proliferation and apoptosis in human brain organoids. **(A)** Cerebral organoids response to tranylcypromine treatment were tested at three concentrations (0.1, 1, 10 µM). **(B,C)** Brain cellular proliferation and apoptosis rates were analyzed by Ki67 and scaspase3 **(C)** expression. Scale bars: 200 µm.

We further utilized the cerebral organoid model to evaluate the neurotoxicity of tranylcypromine. The half-maximal inhibition concentration (IC50) of tranylcypromine was 1 µM, which was calculated by a wide range of doses tranylcypromine treatment on cerebral organoids (Figure S1 in Supplementary Material). Thus, concentrations 10 times more or less than 10 µM (0, 0.01, 1, 10 µM) were used to determine the effect of tranylcypromine on cerebral organoid for further evaluation. After 24-h tranylcypromine treatment, brain organoids resulted in dose-dependent growth inhibition. The cerebral organoids at 0.01 µM tranylcypromine did not show much structural damage compared with control. At the concentration of 1–10 µM, tranylcypromine led to neuroepithelium outgrowth blockage, with significantly thinner brightening epithelium and signs of cell apoptosis (Figure 3A). To investigate the effect of tranylcypromine on brain cellular proliferation and apoptosis, the expressions of proliferation-related gene Ki-67 and apoptotic indicator cleaved caspase 3 were examined. The organoids exposed to tranylcypromine exhibited a decreased expression of Ki-67 in a dose-dependent manner, indicating proliferation inhibition by tranylcypromine (Figure 3B). Moreover, the expression of cleaved caspase 3 significantly at concentrations greater than 1 µM (Figure 3C). Taken together, compared with control organoids, high-dose tranylcypromine-treated organoids displayed markedly structural damage and decreased proliferation as well as induced cell apoptosis to exhibit neurotoxicity.

### Impairment of Astrocytes and Neurons by Tranylcypromine Treatment

**Figure 4:**
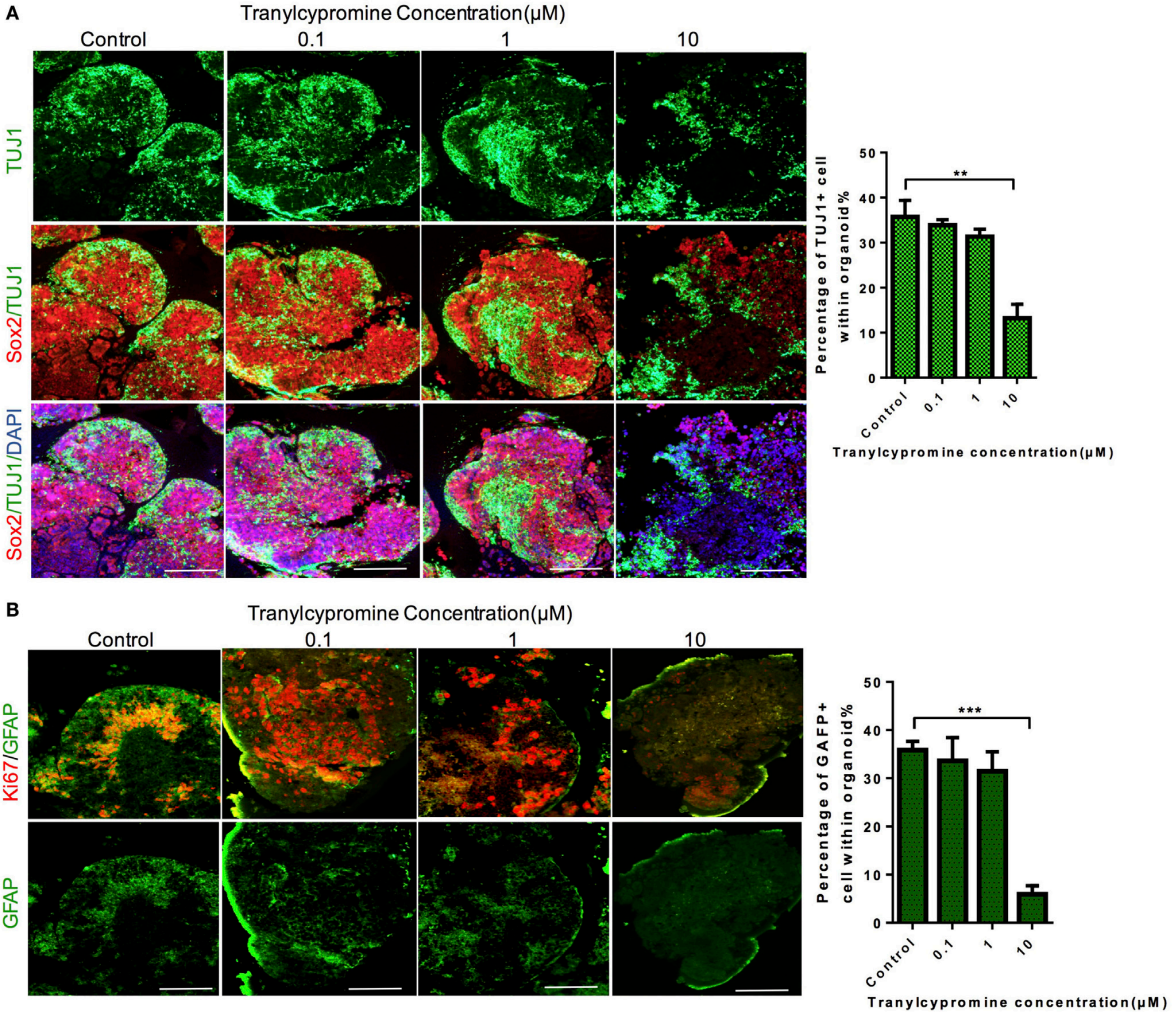
Tranylcypromine treatment impaired neuron and astrocyte of brain organoid. **(A,B)** Immunostaining for the neural progenitor marker Sox2, astrocyte marker GFAP, and neuron marker TUJ1 in human cerebral organoids after treatment with different concentrations of tranylcypromine. Scale bars: 200 µm.

To reveal the mechanisms by which tranylcypromine inhibited proliferation and exhibited neurotoxicity in the cerebral organoid, we detected the expressions of neuron-specific markers TUJ1 and GFAP which stained for neurons and astrocytes separately. In cerebral organoids, although tranylcypromine caused neuron growth attenuated and neuron disarrangement at a relatively high concentration (10 µM), the brain organoids were able to maintain neuron and astrocytes density and arrangement by quantitative analyses at low concentrations (Figures 4A,B). These findings demonstrated that tranylcypromine impaired iPSCs-derived cerebral organoids through reducing neuron and astrocyte at high concentrations.

### Tranylcypromine Inhibited BHC110/LSD1 and Increased the Expression of Histone Di-Methylated K4

Based on the observations that tranylcypromine treatment impaired the proliferation and neuro-specific cells in brain cerebral organoids, we tried to find the signaling regulating these changes after tranylcypromine treatment. As an irreversible MAO inhibitor, tranylcypromine increases serotonergic, noradrenergic activity and augments dopamine transmission (3). Besides, it has been shown to inhibit BHC110/LSD1, as an important chromatin modification enzyme capable of demethylating histones (28).

Histone modifications are very important for normal and pathological development including methylation, acetylation, phosphorylation, ubiquitination, and glycosylation. They can cause gene expression level changes and recruitment of regulatory transcription complexes by chromatin protein structure or interaction function change (33). BHC110/LSD1 has been found to demethylate mono- and dimethyl histone H3 lysine 4 (H3K4) as a FAD-dependent polyamine oxidase which in turn repress gene transcription (28, 34).

**Figure 5:**
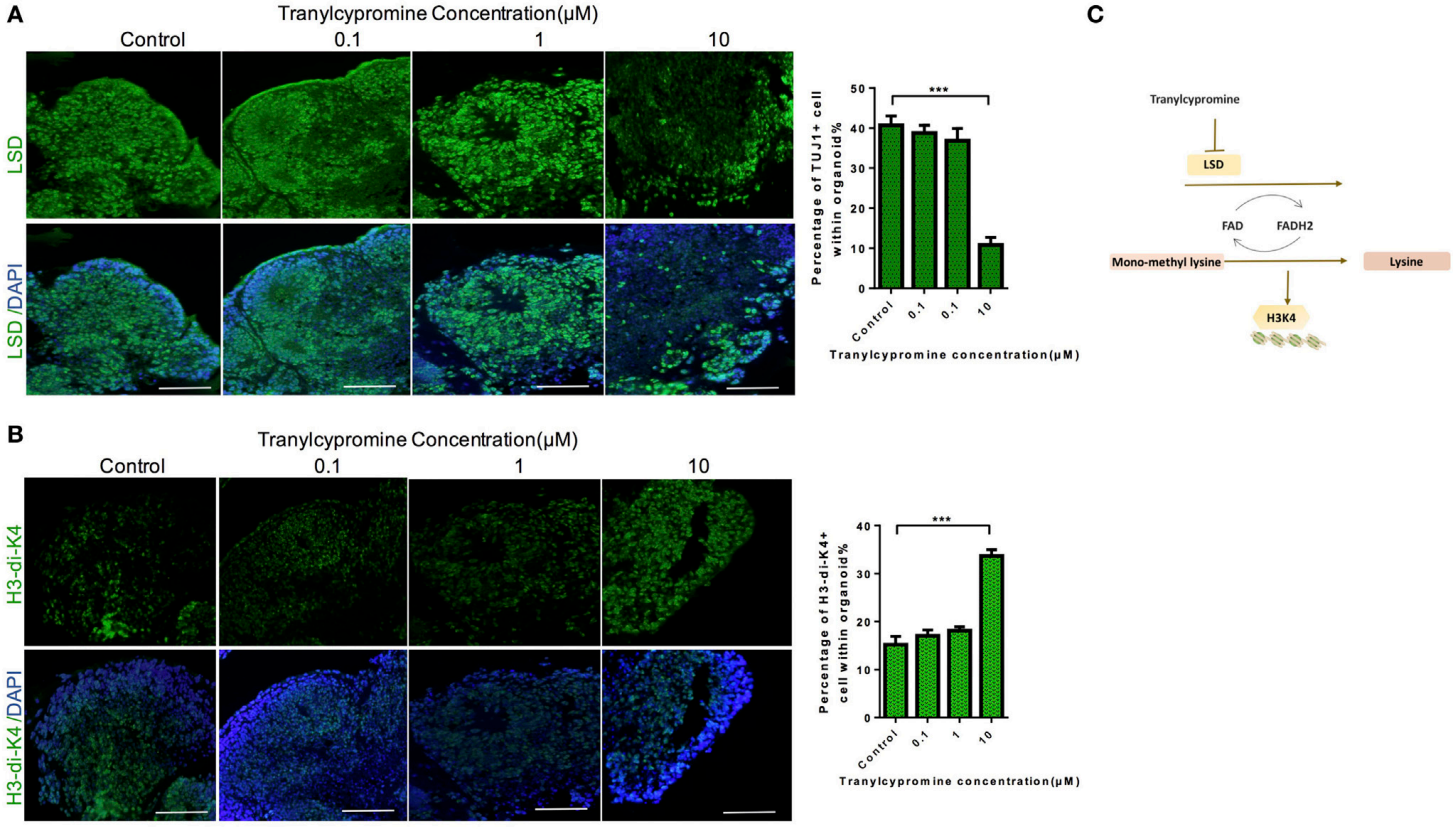
Tranylcypromine inhibited BHC110/LSD1 and increased the expression of histone di-methylated K4. **(A,B)** Immunostaining for the expressions of LSD1 and H3 dimethyl K4 **(B)** after tranylcypromine treatment. **(C)** Schematic diagram showing tranylcypromine inhibits BHC110/LSD1 histone demethylation enzyme.

To find out whether these changes in the cells exposed to tranylcypromine were induced by the inhibition of BHC110/LSD1, we examined the expression of LSD1, which showed marked decrease by tranylcypromine treatment at 10 µM concentration (Figure 5A). The immunohisological analysis also found that H3 dimethyl K4 protein expression was drastically increased at the same concentration in brain organoids compared to the low concentration groups. In particular, the changes of LSD1 and H3 dimethyl K4 seemed not affected at low concentrations (Figure 5B). These results suggested the neurotoxicity of tranylcypromine may be associated with BHC110/LSD1 inhibition (Figure 5C).

## Discussion

Psychiatric disorders, such as schizophrenia, bipolar disorder, and depression, are extremely heritable complex polygenetic neurodevelopmental disorders (1, 35–38). Despite the large market for new psychiatric drug therapies that already exist, the drug discovery for treating psychiatric disorders is stagnant (7, 8). To obtain a precise and proper drug screening model in psychiatry is more urgent than other areas of medicine because it is complex and inaccessible to directly study the human brain. Although the currently available rodent animal models are used a lot for basic drug screening and investigations in neuroscience, they are still time-consuming, costly, and we are unable to study the molecular and cellular effects of these drugs on human brain (39). It is essential to develop an *in vitro* system that recapitulates key characteristics of human brain. With these new hPSC-derived *in vitro* models, it is possible now to investigate precise cellular and molecular mechanisms, the pathways underlying psychiatric drugs and identify new efficacious drugs.

In this paper, we used this *in vitro* brain organoid model to study the effect and neurotoxicity of tranylcypromine. Tranylcypromine is a MAO inhibitor that works by inhibiting the breakdown of norepinephrine, dopamine, and serotonin to treat refractory depression (3). After tranylcypromine treatment, the organoids exhibited a Ki-67 expression decrease and cleaved caspase 3 expression increase in a dose-dependent manner, which indicated inhibited proliferation activity and induced apoptosis. Also, tranylcypromine impaired neural tissue growth in a high dosage with the decreased expression of GFAP and TUJ1 in cerebral organoids. Moreover, treatment of cerebral organoids with tranylcypromine resulted in a global decrease in LSD1 and increase in H3K4 methylation. BHC110/LSD1 is a demethylation enzyme which can demethylate mono- or dimethyl-H3K4 as a FAD-dependent amine oxidase and shared close sequence homology with MAO (29). It has been identified within a number of corepressor complexes together with HDAC1/2, CoREST, and BHC80 (40–42). Former studies have shown that repression of neuronal-specific genes by CoREST required the participation of LSD1 (43). MAO inhibitors were found to be neurotoxic in a concentration-dependent manner in adult rat hypothalamic cell culture (26). And tranylcypromine treatment also showed CNS side effects in clinical therapies (3). The next step to understanding the mechanism of neurotoxicity will be finding out the direct link between BHC110/LSD1 histone demethylation inhibition and tranylcypromine-induced neurotoxicity. Chromatin immunoprecipitation assay to show which loci of the genome H3K4me2 is affected by tranylcypromine is also necessary.

The study opens a new way for neuropsychiatry drug screening. It also allows for the introduction of patient-derived brain organoids as a way to bridge the gap between animal models and human disease. Testing human brain organoids generated from patient-derived iPSCs can link the results with the individual clinical characteristics of each patient. In addition, this technique opens the door for clinical scientists worldwide to use a precise, targeted method to study gene modification, environment invention, and drug therapies tailored to the individual patient as precision research. Further improvements for this iPSC-derived system can be amenable to the maintenance of tissue health and homeostasis to provide a more reliable recapitulation of the *in vivo* developmental process. It should also be able to insert a given genetic or environmental variant, which enables the application of studying the human brain of a defined perturbation experimentally in lab.

In conclusion, we report for the first time that tranylcypromine caused neurotoxicity and BHC110/LSD1-targeted genes suppression on brain organoids. These results show that human brain organoids can be applied as an *in vitro* model for evaluating structural, cellular, and molecular changes of medication.

## Author Contributions

JH, FL, and JC conceptualized and designed the study; FL drafted the initial manuscript; JH, HT, HW, and LL performed the experiments and analyzed the data; RW, JZ, YW, and ZL made substantial contributions to revising the manuscript and figure design. JC is responsible for the overall content.

## Conflict of Interest Statement

The authors declare that the research was conducted in the absence of any commercial or financial relationships that could be construed as a potential conflict of interest.

## Supplementary Material

Figure S1 MTT assay of tranylcypromine-mediated cellular toxicity in the cerebral organoids.
