# Supplementary figures and images for: Tranylcypromine Causes Neurotoxicity and Represses BHC110/LSD1 in Human-Induced Pluripotent Stem Cell-Derived Cerebral Organoids Model
Source: Front Neurol. 2017 Dec 7;8:626. doi: 10.3389/fneur.2017.00626 (PMC5725435; doi:10.3389/fneur.2017.00626)

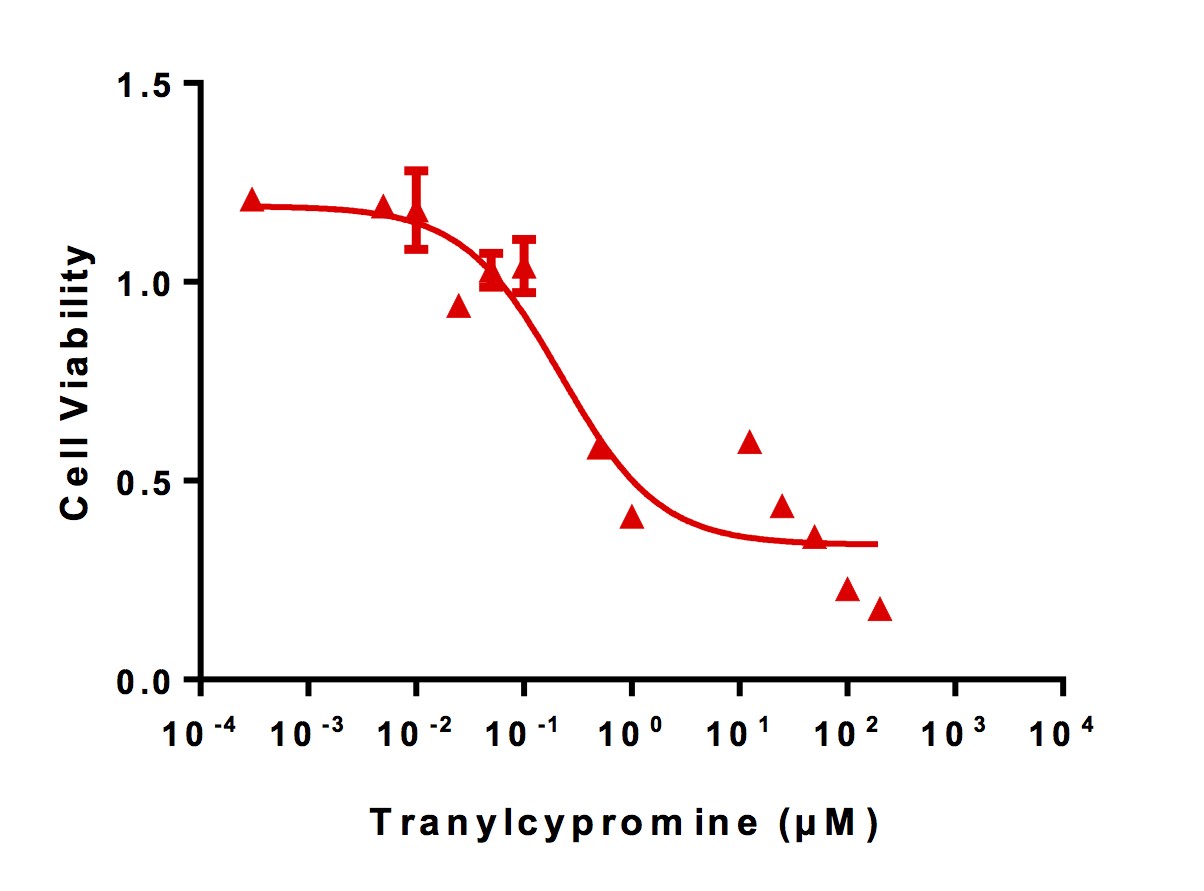

Supplement: Figure S1 — MTT assay of tranylcypromine-mediated cellular toxicity in the cerebral organoids. [file image_1.jpg]
